# Point contact resistive switching memory based on self-formed interface of Al/ITO

**DOI:** 10.1038/srep29347

**Published:** 2016-07-07

**Authors:** Qiuhong Li, Linjun Qiu, Xianhua Wei, Bo Dai, Huizhong Zeng

**Affiliations:** 1 State Key Laboratory Cultivation Base for Nonmetal Composites and Functional Materials, Southwest University of Science and Technology, Mianyang 621010, China; 2 State Key Laboratory of Electronic Thin Films and Integrated Devices, University of Electronic Science and Technology of China, Chengdu 610054, China

## Abstract

Point contact resistive switching random access memory (RRAM) has been achieved by directly sputtering Al electrodes on indium tin oxide (ITO) conductive glasses. The room-temperature deposited Al/ITO shows an asymmetrical bipolar resistive switching (BRS) behavior after a process of initialization which induces a stable high resistive state (HRS). It might be caused by the *in-situ* formation of an ultra-thin layer (≈4 nm) at the interface. By comparison, the Al/ITO device after vacuum annealed exhibits typical symmetrical BRS without an initiation or electroforming process. This can be ascribed to the *ex-situ* thickening of the interfacial layer (≈9.2 nm) to achieve the stable HRS after heat treatment. This work suggests that the self-formed interface of active Al electrode/ITO would provide the simplest geometry to construct RRAM.

Resistive random access memory (RRAM), as one of the most promising candidates of next generation nonvolatile memories, has received much attention^1^,^2^,^3^,^4^. Compared with traditional commercialized flash memories and other emerging nonvolatile memories, RRAM device structure exhibits excellent miniaturization potential because it is universally considered as the simplest memory structure with a switching layer sandwiched between two electrodes^1^,^3^,^5^,^6^. The switching layer is generally the insulator or semiconductor, and its resistance can be reversibly switched between a high resistance states (HRS) and a low resistance state (LRS)^3^,^7^. Sequential stacking of switching layer and top electrode would require different fabrication and patterning process. This inevitably results in the large increase of the complexity of the circuits and the cost of the devices. Very recently, a new RRAM concept of “point contact” between Pt and transparent conductive oxides (TCOs) without the deposition of switching layer has been proposed^8^. Bipolar resistive switching (BRS) in the point contact device is realized through oxygen migration induced by electrical field, including the migration of oxygen ions and oxygen deficiencies in TCOs. It not only breaks the understanding of the “simplest” RRAM sandwiched configuration, but can further increase the storage density utilizing etching/punch-through technique to fabricate “3D” stacking RRAM device.

Unfortunately, a relatively large voltage is necessary to drive the drift of oxygen ions as an initiation process, and thus trigger the transformation from initial conductive state to HRS in point contact devices^8^. Similarly, in TCOs/NiO/Pt structures, ultra-thin oxide layers with unipolar resistive switching can be self-assembled by field-induced oxygen migration at the interface of TCOs/NiO^9^. Actually, the formation of an interfacial layer can be *in-situ* completed between chemical active metal and TCOs during the deposition of metal. Therefore, it would be an opportunity to construct a point contact RRAM by direct deposition of the active electrode on conductive oxide electrode because of the self-formed interfacial layer, as shown in the schematic diagrams of Fig. 1. For comparison, according to the oxygen potential diagram of metal oxides, two types of metal electrodes which are separately relatively inert (Ta) and active (Al, Cr, Sm) to In, are considered in this work. They are directly deposited on indium tin oxide (ITO) at room temperature, and BRS is observed in the active electrodes (Al, Cr, Sm)/ITO structure while no resistive switching is found in the Ta/ITO. Moreover, the properties of interfacial layers could be tailored by heat treatment. We compare resistive switching behaviors of the Al/ITO sample deposited at room temperature and annealed at 400 °C in vacuum. Moreover, we have performed a systematic study on the effect of the two kinds of top electrode materials on the electrical characteristics in metal/ITO structures. The transport mechanisms of them are also discussed.

## Results and Discussion

**Figure 2:**
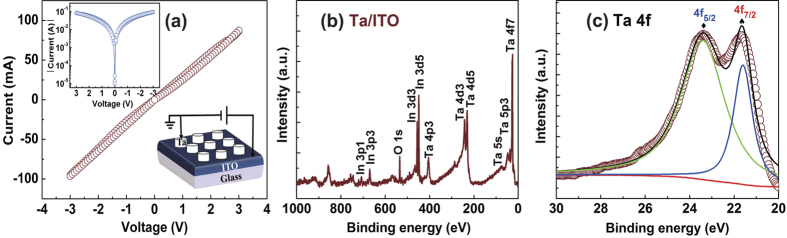
(**a**) *I-V* characteristics of the Ta/ITO sample. Inset: ln(*I*)-*V* curve (top left), the schematic structure for the electrical measurement of Ta/ITO structure (bottom right). The Ta electrode is grounded and the bias voltage is applied on the ITO electrode. (**b**) XPS survey scan of the Ta/ITO interface. (**c**) Ta 4f XPS peaks.

When Ta is used as the top electrode, no resistance switching behavior was observed with Ohmic transport as shown in Fig. 2a. The typical current-voltage (*I-V*) curves of Ta/ITO devices with different bias also show the ohmic behavior (see Supplementary Figure S1). In this case, as Ta is chemically inert considering that the standard Gibbs free energy of formation of Ta_2_O_5_ (−764.4 kJ/mol at 298 K (2/5 Ta_2_O_5_))^10^ is higher than that of In_2_O_3_ (−830.7 kJ/mol at 298 K (2/3 In_2_O_3_))^11^,^12^, it is difficult for it to capture oxygen ions from the ITO substrates. The X-ray photoelectron spectroscopy (XPS) profile analysis also confirmed it. With etching times increasing, the information about In appeared. As shown in Fig. 2b after etching about 1900 s, simultaneously observable Ta, In peaks with close intensity reveal that the XPS survey scan should reflect the chemical composition of Ta/ITO interface. Ta 4f analysis is shown in Fig. 2c. The peak fitting was performed using Shirley background subtraction and Gaussian-Lorentzian function. The binding energy of 21.94 eV and 23.77 eV, corresponding to the Ta 4f_7/2_ and Ta 4f_5/2_ with spin-orbit splitting, is the typical Ta^0^ peak^13^,^14^. There are no any oxides of tantalum, proving that only a pure metal tantalum exists at the interface. Supplementary Figure 2Sa and 3Sb show the integrated depth-XPS peak of Ta/ITO of unannealed and vacuum annealed, respectively. The chemical valence of the interface was unchanged after vacuum annealing. Due to the lack of switching layer, the resistance of the Ta/ITO (about 30 Ω) is determined by Ta film, ITO, and the contact between them. So no resistance switching behavior is generated in this structure as shown in the schematic structure in the inset of Fig. 2a.

**Figure 3:**
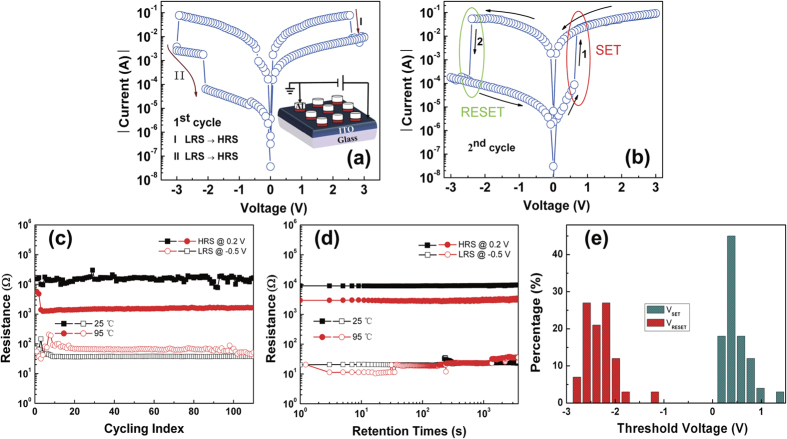
(**a,b**) 1st cycle and 2nd cycle of ln(*I*)-*V* characteristics of the Al/ITO sample. The inset in (**a**) the schematic structure for the electrical measurement of Al/ITO structure. The Al electrode is grounded and the bias voltage is applied on the ITO electrode. The red and green ovals in (**b**) represent the SET (HRS → LRS) and RESET (LRS → HRS) processes, respectively. The arrows and numbers indicate the switching sequences. (**c**) Endurance performance of the device at 25 and 95 °C. (**d**) Retention properties of the device at 25 and 95 °C. (**e**) Distributions of SET and RESET threshold voltage.

In contrast, resistance switching properties of Al/ITO structure deposited at room temperature is demonstrated by the *I-V* curve, endurance test, data retention, and threshold voltage (*V*_th_) distribution as shown in Fig. 3. Before the presence of memory behavior, an initial process is needed shown in Fig. 3a. The pristine devices are initially at LRS with an initial resistance about 40 Ω. It is very close to the Ta/ITO sample. This result suggests the interfacial layer could not be present or the interfacial layer could be conductive. The current increases slowly with the increase of the applied positive voltage on the bottom electrode. When the voltage reaches 2.6 V, the current decrease abruptly and the resistance increases by one order of magnitude than the initial state (step I), as shown in Fig. 3a. This suggests that the transport of the device may be dominated by the interface. Considering that the drift direction of oxygen ions is not to the Al top electrodes at positive voltage, the enhancement of the resistance could be due to the thermal effect at a large current which could promote partial oxidation of Al. Under the negative voltage sweeping region, the resistance does not show memory effect, and recovers to the initial state, indicating that the interfacial layer is not stable. While the bias sweeps to −2.9 V, the resistance switches to an intermediate resistive state, and at last at −2 V it reaches to a HRS with 5 × 10^4^ Ω (step II). It is reasonable to propose that a lot of oxygen ions migrate to the Al top electrodes across the interface. Under this case, the thickness and the oxygen content of the interfacial layer would be enhanced, which results in the arrival of final HRS. Moreover, the HRS is stable and shows memory effect. During the second voltage sweep (0 → 3 → 0 → −3 → 0 V), the devices exhibit a set process from HRS to LRS at about 0.6 V, and a reset process from LRS to HRS at about −2.4 V as shown in Fig. 3b. BRS can also be found in Cr/ITO and Sm/ITO samples as shown in Figure S4. Similar to the unannealed Al/ITO sample, the BRS needs an initiation process. The two metallic electrodes have a lower standard Gibbs free energy for formation of the corresponding oxides than In_2_O_3_, such as Cr (Cr_2_O_3_: −1058.1 kJ/mol (2/3 Cr_2_O_3_)) and Sm (Sm_2_O_3_: −1734.6 kJ/mol (2/3 Sm_2_O_3_)) at 298 K^11^.

A stable BRS behavior has been obtained over 100 cycles with the resistance ratio (*R*_OFF_/*R*_ON_) more than 10^2^ in the as-fabricated Al/ITO structure. Besides, no significant changes in the resistance magnitudes for more than 10^3^ s can be observed (Fig. 3d). Furthermore, the endurance characteristic and data retention of Al/ITO sample are tested at 25 and 95 °C in the sweep measurement mode as shown in Fig. 3c,d. However, there is a slight degradation of HRS during the measurement of endurance and retention at 95 °C. The statistical electrical performance of the device is summarized in the histograms of set voltage (*V*_SET_) and reset voltage (*V*_RESET_) values for the Al/ITO sample after 100 cycles as shown in Fig. 3e. *V*_SET_ has about 40% probability to be at 0.4 V, and *V*_RESET_ has about 75% probability to be between −2.2 and −2.6 V. It indicates an asymmetric distribution of *V*_th_ between *V*_SET_ and *V*_RESET_.

**Figure 4:**
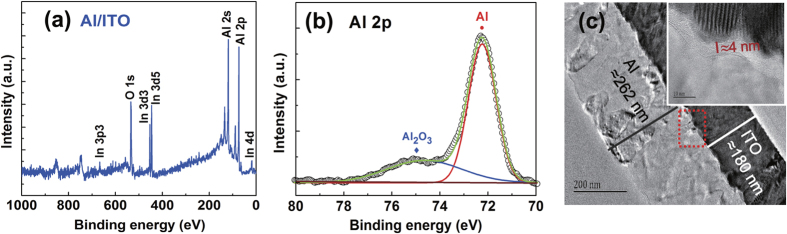
(**a**) XPS survey scan of the Al/ITO interface. (**b**) Al 2p XPS peaks. (**c**) Cross-sectional TEM images of pristine Al/ITO structure, Inset: the HRTEM image of the interface of pristine Al/ITO.

In our previous reports, effect of the interface layer between oxide and Al electrode on resistive switching of Al/NiO/ITO should be considered^15^,^16^. Similarly, there should be an ultra-thin AlO_x_ layer existed at the Al/ITO interface due to the standard Gibbs free energy of formation of oxides of metals. The ability of Al to absorb oxygen ions (Al_2_O_3_: −1582.9 kJ/mol (2/3 Al_2_O_3_)) at 298 K^11^,^17^ is greater than In. XPS profile analysis and cross-sectional high-resolution transmission electron microscopy (HRTEM) also confirmed it. Al and In peaks with close intensity can be observed in the XPS survey after etching about 2200 s as shown in Fig. 4a. Different from Ta peaks in Ta/ITO, the Al 2p peaks can be fitted by two parts in Fig. 4b, locating at 72.3 and 74.4 eV which correspond to the metallic Al−Al bonding and Al−O bonding of the Al_2_O_3_, respectively^18^. The cross-sectional HRTEM demonstrates that obvious diffusion of oxygen atoms happened at the interface of Al/ITO device as depicted in Fig. 4c. The AlO_x_ interface layer is about 4 nm thickness and has an amorphous structure. Nevertheless, the room-temperature pristine Al/ITO structures show conductive not insulating. There might be two reasons for the abnormal resistance. One is oxygen deficiency in AlO_x_ interface layer, which also can be considered as Al rich, inducing the formation of the conducting path. Another is attributed to the ultra-thin thickness of AlO_x_ interface layer, which could provide the possibility to tunnel across the interface. These also could be the reasons why the initial process is needed before the presence of memory behavior in the room-temperature deposited Al/ITO structures. After the initial process, the structures can be switched to a stable HRS, due to the oxygen supplies to the interface under an external electric field. It would suppress the effects of the two factors. The initial process from LRS to HRS is opposite to the electroforming process in which a large voltage induces the device from HRS to LRS^19^. It is also different from the initial process in the Pt/TCOs structures with a relatively large voltage. In this work, the bias magnitude of the initial process is equivalent to that of BRS behavior in Fig. 3b. It might be associated with self-assembled interfacial layer due to interface chemical reaction^15^,^17^,^20^.

**Figure 5:**
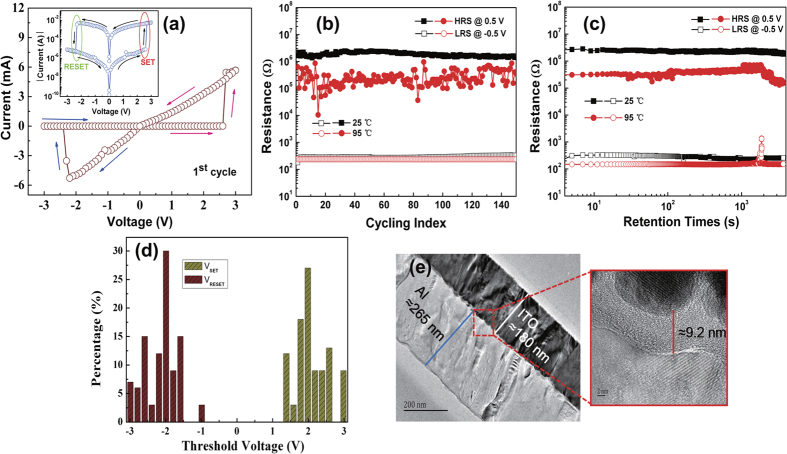
(**a**) Electrical properties of the Al/ITO sample after vacuum annealed. Inset: the ln*(I*)-*V* curve. The Al electrode is grounded and the bias voltage is applied on the ITO electrode. The red and green ovals represent the SET and RESET processes, respectively. The arrows indicate the switching tendency. (**b**) Endurance performance of the annealed sample at 25 and 95 °C. (**c**) retention characteristics of the annealed sample at 25 and 95 °C. (**d**) Distributions of SET and RESET threshold voltage. (**e**) Cross-sectional TEM images of the interface of annealed Al/ITO.

The room-temperature fabricated Al/ITO devices show the BRS behaviors with a large working current. It will cause the increase of power consumption and the damage of devices. We infer that it might be caused by the ultrathin AlO_x_ interface layer and the requirement of the initiation process. Interface reaction can be accelerated at a higher temperature, which should be expected to enhance the thickness of the interfacial layer. Therefore, the room-temperature fabricated Al/ITO devices were *ex-situ* annealed at 400 °C for 4 h in vacuum. Figure 5a–c shows the resistance switching properties, including the *I-V* characteristic, endurance and data retention of the devices after vacuum annealing. It is worth noting that the BRS behaviors have occurred without an initiation or electroforming process. And the devices are initially in the high resistance state (HRS) with initial resistance about 10^6^ Ω (@ 1 V) as shown in Fig. 5a. The endurance characteristic and data retention of the vacuum annealed sample have been tested at 25 and 95 °C as shown in Fig. 5b,c. The *R*_OFF_/*R*_ON_ of the Al/ITO sample after annealing increases by almost two order of magnitude and reaches 10^4^ at 25 °C. Endurance test can be stably obtained over 100 cycles, as shown in Fig. 5b. Moreover, Fig. 5c shows the data retention of vacuum annealed Al/ITO sample over 10^3^ s. But the retention time insufficient as a memory from the conventional point of view^21^. It may be caused by the rough interface due to the rough surface of ITO substrate as shown in the HRTEM results as shown in Figs 4c and 5e. It would give a negative influence on the transport properties of the ultra-thin interface layer. Data retention of the self-formed interface RRAM could be improved by achieving a sharp interface on an atomically smooth substrate surface. Thus, it needs further investigation. The histograms of *V*_th_ is shown in Fig. 5d, indicating a symmetrical distribution of the *V*_th_ between *V*_SET_ and *V*_RESET_. For un-annealed Al/ITO, BRS needs an initiation process due to its unstable interface. In this case, the distribution of non-homogeneous defects such as oxygen vacancies might exist. The asymmetrical BRS behavior could be explained by its asymmetric stacking layers structure^22^. After annealing, the defects could distribute throughout the interface more homogeneously due to stronger diffusion ability at higher temperature. It might induce the symmetrical operation voltages of the vacuum annealed sample. Thus, the *ex-situ* thermal formation process can directly achieve a stable HRS in which the interfacial layer is more insulating than that through the electrical initiation process in the room-temperature fabricated sample. It can be confirmed by the cross-sectional HRTEM images of the interface between the Al electrode and ITO film shown in Fig. 5e. The thickness of the interface layer of about 9.2 nm is significantly enhanced after annealing.

**Figure 6:**
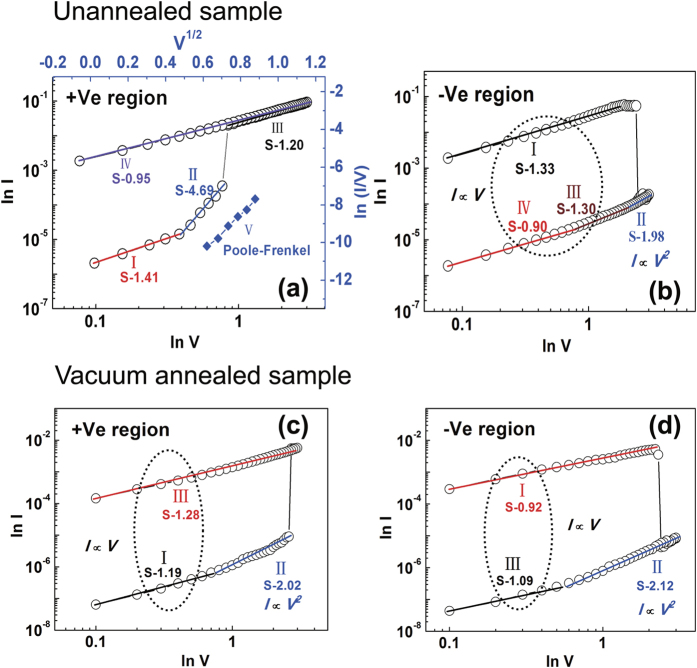
Double logarithmic plotting of *I*-*V* switching curves in Fig. 3b of the unannealed Al/ITO sample: (**a**) set process. (**b**) reset process. Double logarithmic plotting of *I*-*V* switching curves in Fig. 5a of the vacuum annealed Al/ITO sample: (**c**) set process. (**d**) reset process.

Compared with unannealed sample, the annealed sample shows a lower working current and a higher *R*_OFF_/*R*_ON_. More interestingly, a more symmetrical resistance switching behavior without an initiation process has appeared. For a better understanding of the conduction mechanisms of the BRS behaviors in Fig. 3b, the *I-V* curves are fitted with appropriate charge transport models and re-plotted with double logarithmic scale during the set and reset process in Fig. 6a,b, respectively. In RRAM operation, the Ohmic contact relationship is described by *I*(*V*) = *aV* while the space charge limited current (SCLC) is represented as *I*(*V*) = *aV* + *βV*^2 ^23,24,25. In the positive voltage region, the slopes of the linear fitting curves at LRS and HRS (<0.38 V) are about 1 correspond to Ohmic conduction behavior. It is typically due to the formation of conductive filaments in the interfacial layer. At high voltages of HRS (>0.38 V), the slope is much larger than 1. It can be fitted by In(*I*/*V*) ∝ *I*^1/2^ as shown in the blue lines of Fig. 6a. It seems to be governed by Poole-Frenkel (PF) emission caused by the thermal effect and the trapping or detrapping^25^,^26^,^27^,^28^. The PF emission expression can be seen in the Equation 1 29,30,31,
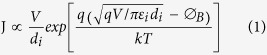


Where *J* is the current density, *V* is the voltage, *d*_*i*_ is the thickness, ε_*i*_ is the permittivity, *φ*_*B*_ is the barrier height, *k* is the Boltzmann constant, and *T* is the temperature. The PF mechanism indicates the existence of a defective AlO_x_ layer since it is due to emission of trapped electrons from an insulator layer. Oxygen vacancies that can create a donor level near the conduction band are the source of electron carriers in n-type semiconductors^1^. But there are a relatively high density of defects and traps in the switching layer. The conduction mechanisms should be determined by the interaction of the injected carriers and defects or trap centers^27^. When the bias is high enough to dissociate AlO_x_ into Al and O^2−^ during the set process, the thickness of AlO_x_ layer decreases, and the current accordingly increases abruptly as shown in Fig. 6a branch II. This is also why a very low set voltage is found. Similar results have been observed in Al/Pr_0.7_Ca_0.3_MnO_3_/Pt structure^29^. Moreover, temperature dependent resistance values of the unannealed Al/ITO sample is measured as shown in Supplementary Figure S5. The resistances were measured at the voltage of 0.2 V in a temperature range of 300~400 K. In the inset of the Figure S5a (see Supplementary Figure S5a), the activation energy obtained from the slope of the Arrhenius plot is 0.13 eV. The activation energy is compared with first ionization of oxygen vacancies (≈0.1 eV), with the conclusion that the first ionization of oxygen vacancies is responsible for the conduction at HRS^32^. Additionally, the temperature dependence of the electrical transport property of the device at HRS shows a semiconducting behavior. It can further support that oxygen vacancies or oxygen ions are mobile ions.

In the negative voltage region, the slopes of the LRS and HRS (<0.77 V) are about 1, and also obeying the Ohmic law. At high negative voltages of HRS (−0.77~−3 V), it follows the square dependence on the voltage, corresponding to Child square low (*I* ∝ *V*^ 2^), indicating that the switching from LRS to HRS is controlled by SCLC which is known to be triggered by oxygen deficiency in the self-formed interface layer. When the density of thermally generated free carriers inside the AlO_x_ interface layer is greater than that of the injected electrons from the ITO, *I* ∝ *V* correlations (Ohmic behavior) are observed^20^,^33^. Once the density of injected electrons gradually exceeds the thermally generated carriers in the film, the conductive mechanism will turn into SCLC. Thus the current in this process is dominated by the injected electrons^34^,^35^. As the negative bias increases, the occupied trap will discharge the electrons. When the voltage reaches the trap-filling-limit voltage (*V*_TFL_), electrons detrap from the majority of occupied traps, the current reduces quickly and the device switches from LRS to HRS^27^. When the negative voltage reduces from −3 to 0 V, a majority of traps are emptied. The device maintains HRS. For the annealed sample, the *I*-*V* curves in the positive and negative voltage regions of Fig. 5a are re-plotted in a log-log scale in Fig. 6c,d, respectively. The transport mechanisms at the HRS state during the set and reset processes are both SCLC, which might lead to a symmetrical switching. The different mechanisms of the two devices might be ascribed to their different initial states. The unannealed devices need an initiation process under electrical stimuli because they are initially conductive with more defect states, and higher oxygen vacancy concentration. Conversely, the annealed devices exhibit an initiation-free BRS behavior with a large *R*_OFF_/*R*_ON_. It is due to the more insulating interfacial layer.

**Figure 7:**
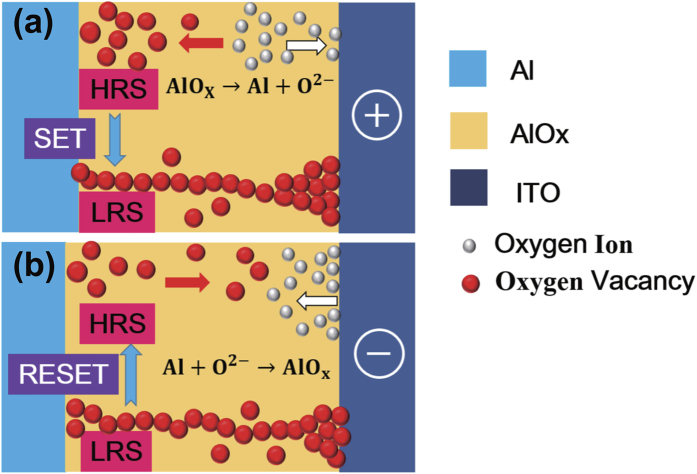
Schematic diagram of the driving mechanism for Al/ITO memory device: (**a**) SET process. (**b**) RESET process.

Considering the existence of the defective AlO_x_ interface layer and the migration of oxygen ions (O^2−^) and Vo^2+^, the resistive switching behaviors of the Al/ITO structure can be demonstrated as the schematic diagram as shown in Fig. 7. In the initiation process, O^2−^ and Vo^2+^ are regulated by the electric field, which makes the AlO_x_ interface layer to acquire lower density of defects and to arrive at a stable HRS. When the negative bias is applied on the Al electrode, the O^2−^ are repelled form the AlO_x_ interface to ITO, and the dissociation process as AlO_x_ → Al + O^2−^, thus the Vo^2+^ are accumulated to form the conductive filaments in the local region of the AlO_x_ interface layer which results the SET process as shown in Fig. 6a. When the positive bias is applied on the Al electrode, the O^2−^ are extracted back to the AlO_x_ interface and OFF state is achieved. In this process, the ITO films act as defect reservoir or source of O^2−^, causing the partially oxidized of the active Al electrodes^19^,^20^,^36^,^37^,^38^. It can be expressed as Al + O^2−^ → AlO_x_, leading to a more uniform interface layer to act as the switching layer. The Area dependence of resistance values in the ON and OFF states as shown in Supplementary Figure S6. Both of them are inversely proportional to the device area, indicating that the change is attributed to the field-induced change of the self-formed AlO_x_ at the interface of Al/ITO over the entire electrode area in this point contact RRAM device. Thus, it can be inferred that the generation of conduction channels are induced by the migration of oxygen ions and oxygen vacancies of the point contact Al/ITO devices owing to the defective AlO_x_ layer.

## Conclusion

In summary, point contact resistive switching memory based on active Al electrode/ITO structure is deposited by sputtering at room temperature. The structure shows the asymmetrical BRS behavior with a very low set voltage after an initiation process. The *I-V* curves indicate that the conduction behaviors in HRS and LRS can be well fitted by PF emission and SCLC mechanism respectively. We also provide the direct evidences of the existence of self-formed switching layer (AlO_x_) due to the interface diffusion through the HRTEM and XPS. Additionally, the vacuum annealed Al/ITO exhibits the symmetric switching behavior without an initiation or electroforming process due to the thickening of interfacial layer. It also indicates that resistive switching behaviors can be tuned in active metal/ITO structures by designing the interface properties like the thickness, the concentration and distribution of defects.

## Methods

Al and Ta point electrodes were deposited on ITO substrates (SnO_2_ : In_2_O_3_ about 1: 9, ITO thickness 180 ± 20 nm, resistivity 5 × 10^−4^ Ω ∙ cm) at room temperature by direct current magnetron sputtering using Al and Ta targets in Ar gas, with the gas flow is 40 sccm, the working gas pressure of 0.4 Pa, and the base pressure of 1.5 × 10^−4^ Pa. For the preparation of the RRAM devices, the ITO glasses were cut into square plates (25 mm × 25 mm). Before the deposition, the substrates were immersed sequentially in isopropyl alcohol, acetone and distilled water by using an ultrasonic bath, each for about 15 minutes. At last, the ITO substrates were blown dry with nitrogen and placed in a holder into the chamber. The thickness and diameter of top electrodes were respectively about 250 nm and 500 μm using metallic perforated masks.

The electrical properties of the device were measured using a Keithley 2400 Source Meter at room temperature. During the test procedure of electrical characterization, top electrodes were grounded and a bias voltage was applied on the ITO electrode. The chemical composition of the film was analyzed by X-ray photoelectron spectroscopy (XPS) with monochromatic Mg Ka radiation (1253.6 eV). The spectra was recorded in the range of 0~1120 eV with a step of 0.5 eV after the samples were sputter-etched with Ar^+^ ions with various sputtering etching times. The binding energy scale of the spectra was calibrated with respect to C 1s signal (285.0 eV) corresponding to adventitious carbon present on the sample surface. Moreover, the interface properties were investigated by cross-sectional high-resolution transmission electron microscopy (HRTEM, JEOL 2100 F) operated at 300 kV. The specimens were prepared by focused ion beam micro-nano processing.

## Additional Information

**How to cite this article**: Li, Q. *et al*. Point contact resistive switching memory based on self-formed interface of Al/ITO. *Sci. Rep.* **6**, 29347; doi: 10.1038/srep29347 (2016).

## Supplementary Material

Supplementary Information

## Figures and Tables

**Figure 1:**
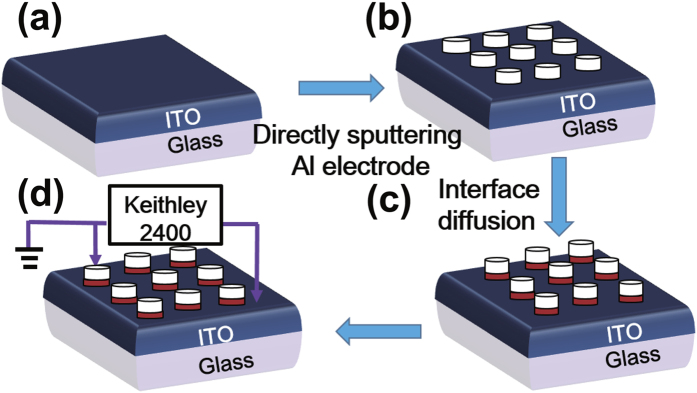
Schematic diagram of point contact RRAM of Al/ITO: (**a**) ITO/glass substrate. (**b**) Deposition of Al electrode. (**c**) Formation of the interface layer caused by diffusion. (**d**) Electrical properties measurement of the device.
