# Supplementary material for: Point contact resistive switching memory based on self-formed interface of Al/ITO
Source: Sci Rep. 2016 Jul 7;6:29347. doi: 10.1038/srep29347 (PMC4935939; doi:10.1038/srep29347)
Supplement: Supplementary Information [file srep29347-s1.doc]

Supplementary Information for

**Point contact resistive switching memory based on self-formed interface of Al/ITO**

Qiuhong Li ^1^, Linjun Qiu ^1^, Xianhua Wei ^1^*, Bo Dai ^1^, and Huizhong Zeng ^2^

^1^State Key Laboratory Cultivation Base for Nonmetal Composites and Functional Materials, Southwest University of Science and Technology, Mianyang 621010, China.

^2^State Key Laboratory of Electronic Thin Films and Integrated Devices, University of Electronics Science and Technology of China, Chengdu 610054, China.

*Corresponding author. Tel: +86 159 0821 4561, Fax: +86 816 2419 492

E-mail: [weixianhua@swust.edu.cn](mailto:weixianhua@swust.edu.cn)





**Figure S1.** Representative *I*-*V* curves of the Ta/ITO devices with different voltage range. (a) -1～1 V, (b) -2～2 V, (c) -3～3 V, (d) -4～4 V, (e) -5～5 V. The inset shows ln(*I*)-*V* characteristics of the Ta/ITO sample.

**
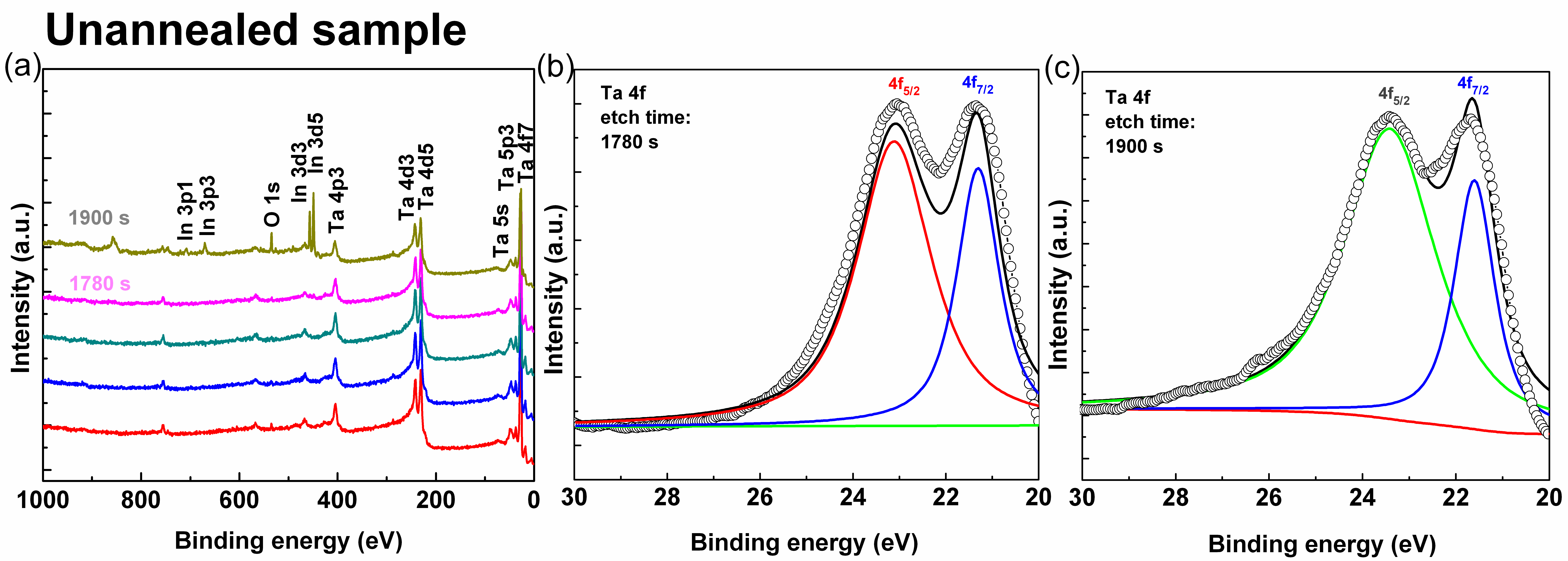
**

**Figure S2.** (a) The full XPS spectra with different etching time of unannealed Ta/ITO sample. The XPS spectra of Ta 4f from the surface of Ta/ITO sample with etching times (b) 1780 s, (c) 1900 s.

**
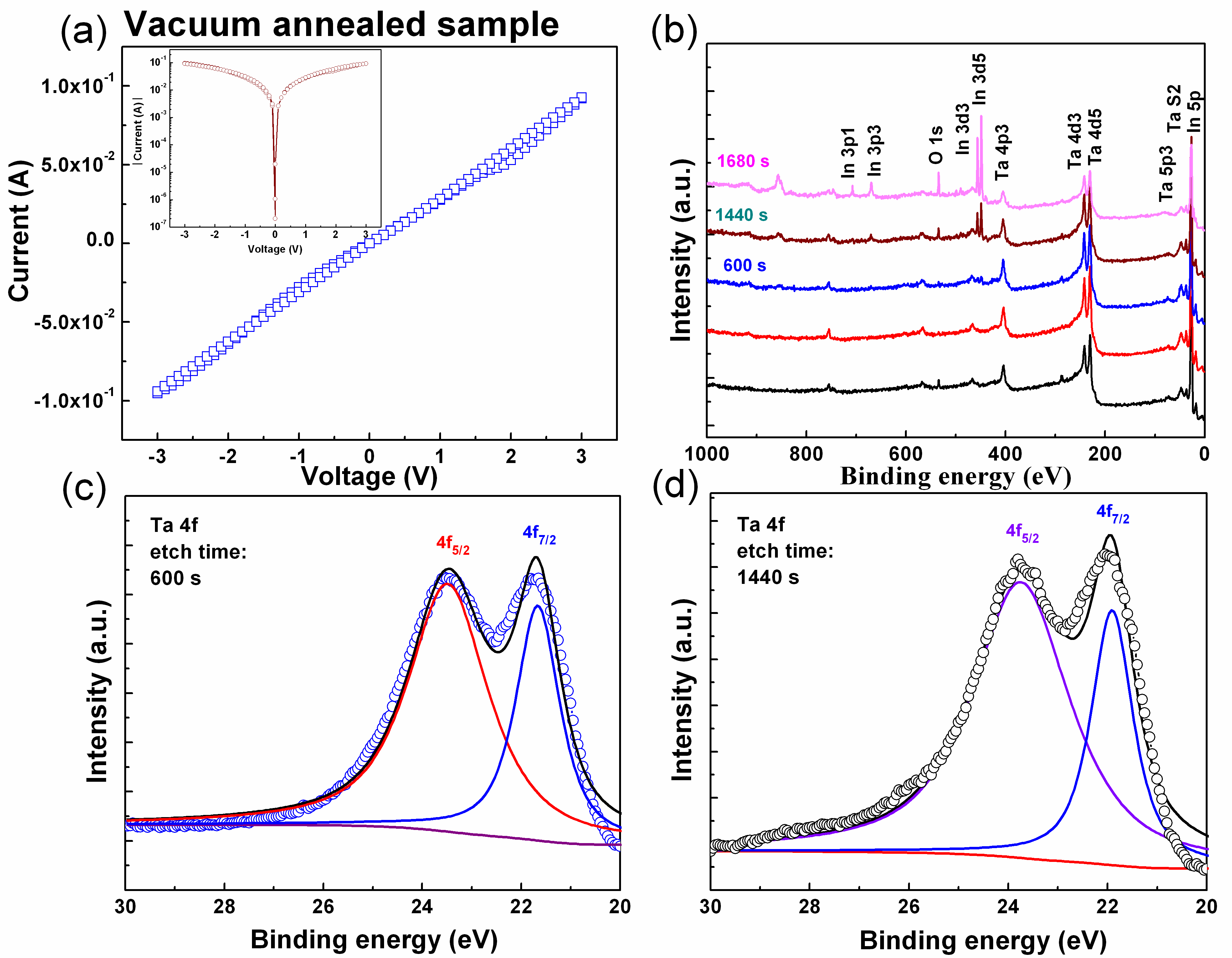
**

**Figure S3.** (a) The *I*-*V* curve of the Ta/ITO sample after vacuum annealed. (b) The full XPS spectra with different etching time of Ta/ITO sample. XPS spectra for Ta 4f from the surface of Ta/ITO sample with etching times (c) 600 s, (d) 1440 s.


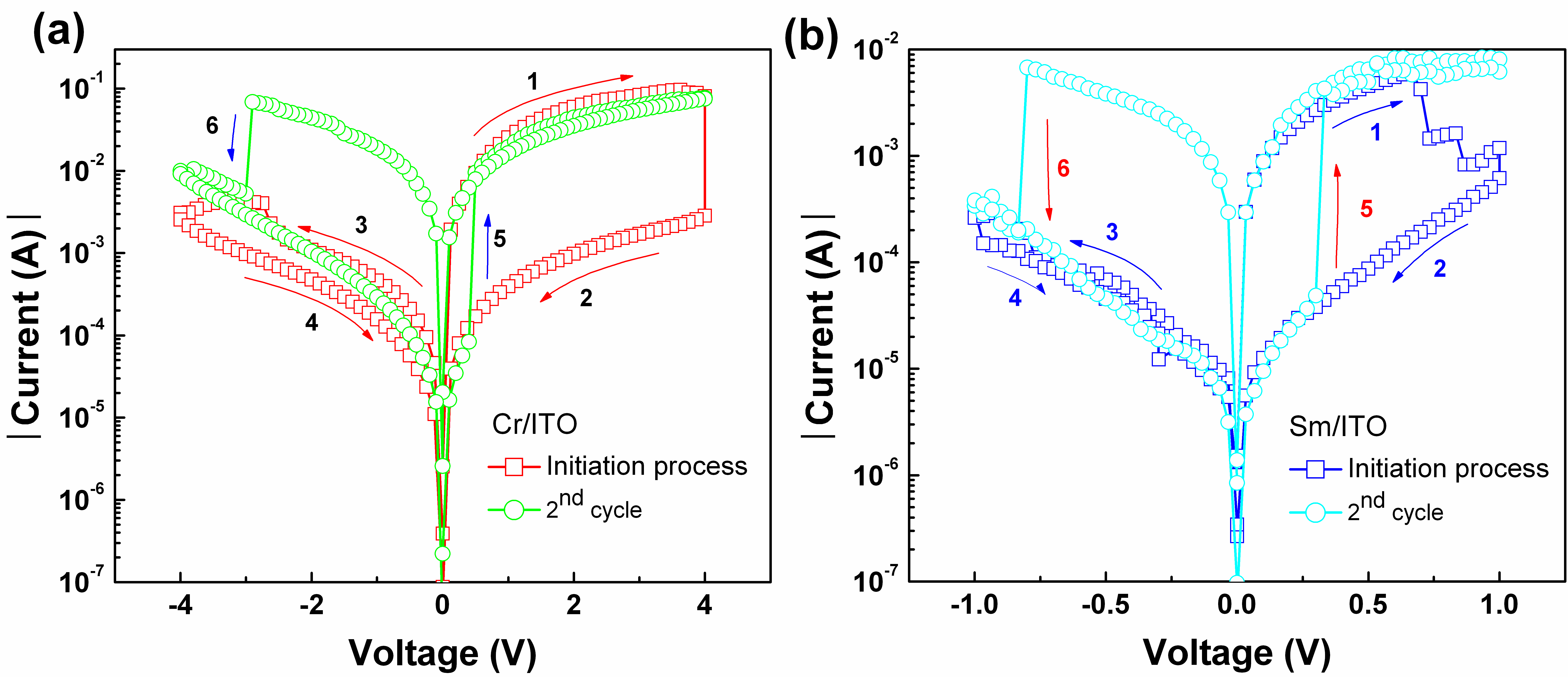


**Figure S4.** The ln(*I*)-*V* characteristics of the unannealed samples: (a) Cr/ITO, (b) Sm/ITO.


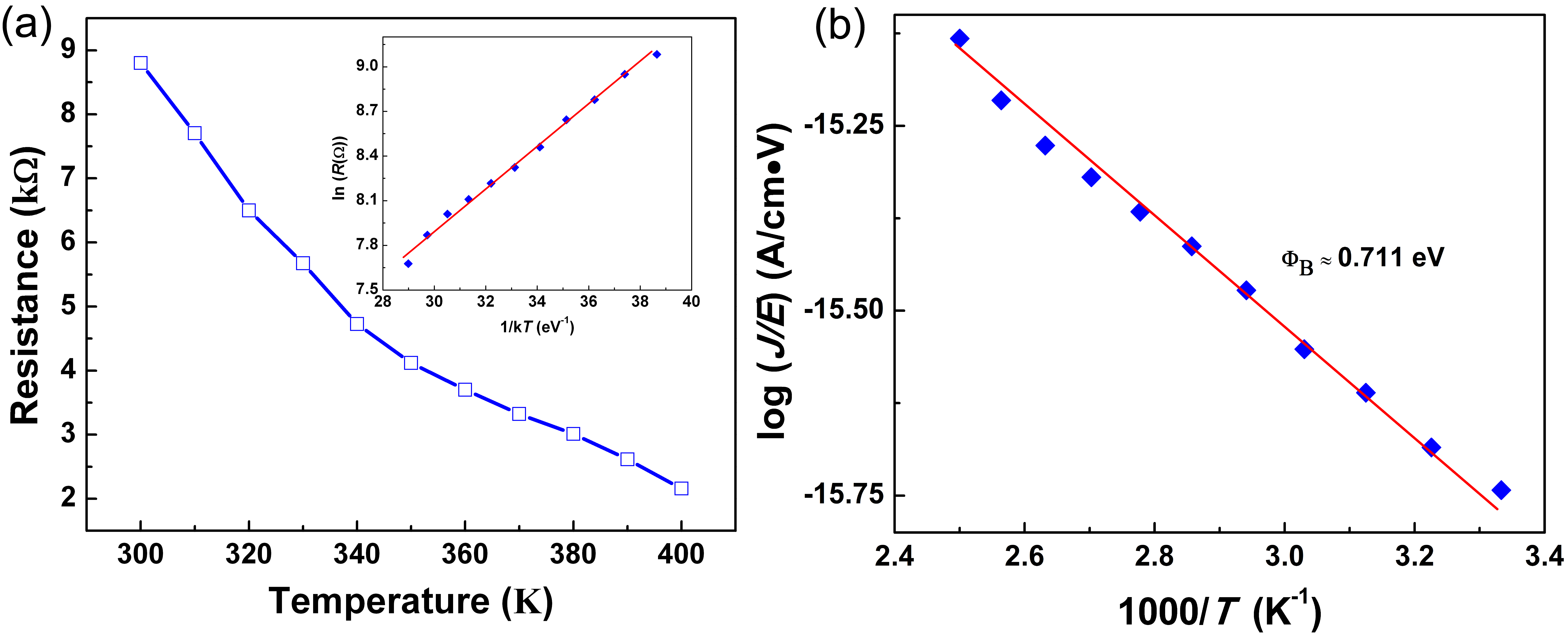


**Figure S5.** (a) Temperature dependence of resistance in HRS. The inset is the Arrhenius plot of the resistance temperature data in HRS. Here, R_OFF_ is extracted at 0.2 V. (b) The plot of the extrapolated value of log (*J*/*E*) ∝ 1000/*T*.


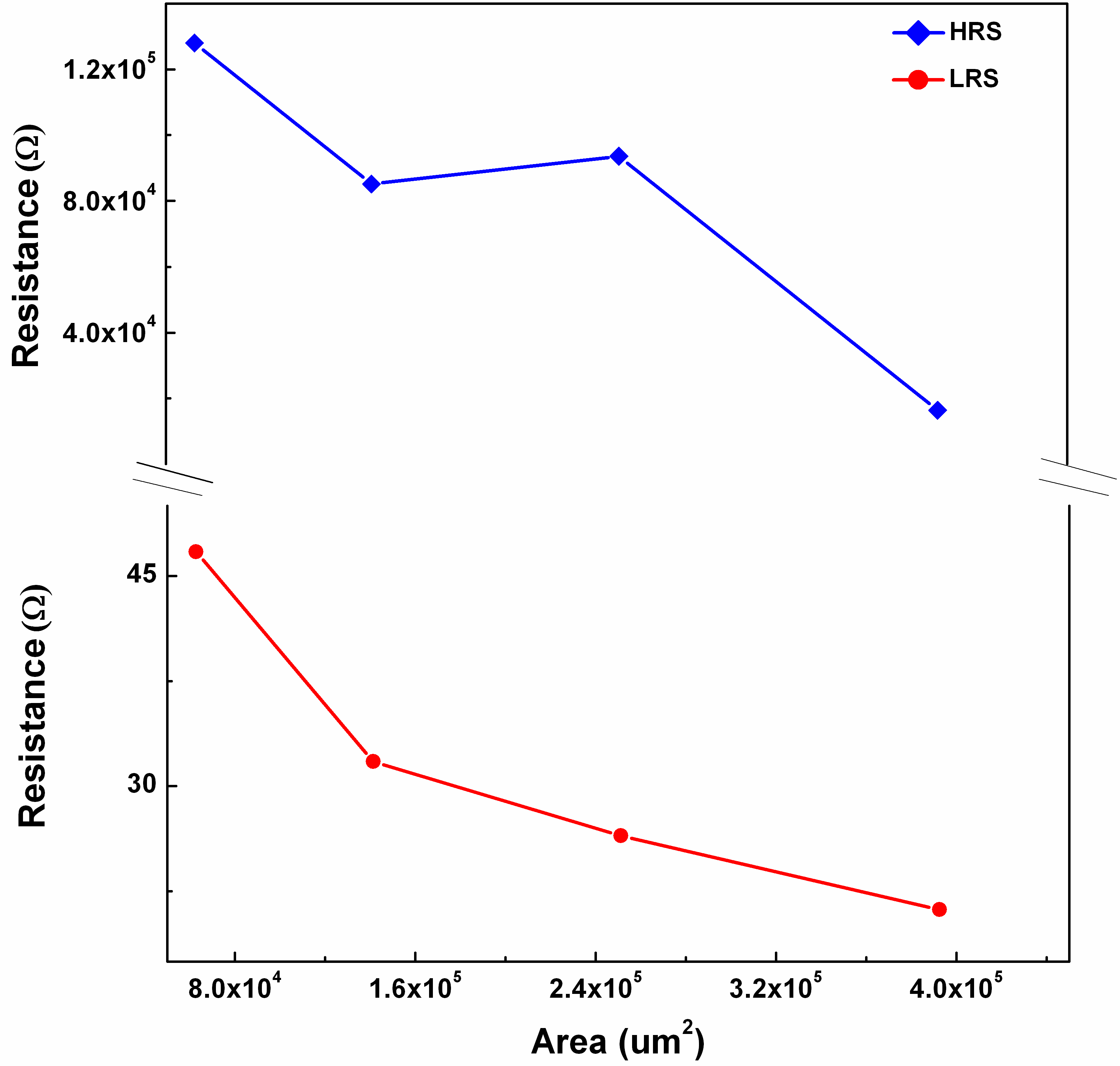


**Figure S6.** Area dependence of resistance in the ON and OFF states.
